# The Treatment of Cirrhosis

**Published:** 1900-04-28

**Authors:** 


### THE TREATMENT OF CIRRHOSIS.

In the Lumelian Lectures which have just been delivered before the Royal College of Physicians, Dr. Cheadle has dealt mainly with the three chief forms of cirrhosis of the liver—atrophic, hypertrophic, and syphilitic.

In atrophic cirrhosis the fibrous invasion is described as spreading along the main branches of the interstitial tree, embracing groups of lobules and compressing them, and thus causing atrophy and destruction of cells. The new fibrous growth between the enclosed groups of lobules contracts and, drawing in the surface at these points, causes hobnailing of the surface and shrinkage of the whole organ, which becomes much smaller than normal, tough and hard, and by obstructing portal circulation causes ascites, hæmorrhage from the stomach and bowels; jaundice is rare. The downward course of the malady after it can be recognised is rapid.

In hypertrophic, or "biliary" cirrhosis, the liver is increased in size and weight, the fibrosis affects the smaller twigs rather than the main branches, embracing single lobules, and even single cells, instead of groups of lobules, and is accompanied by a curious development of biliary canaliculi at the periphery of the lobule. The liver is large, hard, and smooth, or only slightly granular. Ascites is absent or very slight, jaundice is constant, and the course is apt to last some years.

Syphilitic cirrhosis is characterised by the presence of broad bands of fibrous tissue separating isolated masses of liver tissue and causing destruction of hepatic cells. The result is that the organ is often much deformed by the contraction of these fibrous bands, so that we get a hard liver, usually enlarged, sometimes contracted, with an uneven, bossed, or slightly irregular surface.

We have not space to follow Dr. Cheadle in the many interesting points he raised in regard to these different forms of cirrhosis, so we pass at once to the question of treatment. This resolves itself chiefly into an attempt to preserve the *status quo* so far as the liver is concerned, and to relieve any difficulties which may result from the hepatic inefficiency, for no remedy will remove the fibrous tissue which has choked the liver cells, and no drug will restore the cells thus destroyed. The only condition in which the invading agent can be, to some extent, removed by treatment is in the early stage of syphilitic cirrhosis. With regard to the prevention of the increase of fibrosis, absolute abstention from alcohol and all stimulating ingesta is, of course, the prime necessity, and is as essential in the syphilitic and malarial cases as in those of alcoholic origin. There is, however, this reservation to be made, that when the last stage of exhaustion and emaciation is reached, total deprivation of alcohol may hasten the end. Again, foods which tax the physiological function of the liver, such as those which are rich in fat and sugar, should be prohibited or given in reduced quantity, while pancreatised preparations, or pancreatin in keratin capsules, given along with food, are useful aids in cases in which wasting and debility are met with.

Mild laxatives, such as salts of soda and magnesia, together with rhubarb or cascara, are useful in relieving portal congestion, and to them bitter tonics may be added on occasion. There are, however, three drugs of especial value—mercury, iodides, and digitalis. The two first-named should not be restricted to syphilitic cases; nor should digitalis be given only when there is obvious heart disease.

The main interest, however, in the treatment of cirrhosis centres in the ascites with which it is so often accompanied, and this not merely because the occurrence of ascites shows how far the disease has already gone, but because it is in itself a serious cause of interference with circulation, digestion, and elimination. Whatever differences may exist as to the mode in which the fluid is to be removed, there is no question as to the necessity of removing it. There is no more painful picture of hopeless disease and helpless therapeutics than these cases of ascites left unrelieved in their increasing exhaustion and distress. As to the older methods by purgatives, diuretics, and restriction of fluids, it may be said that, although a few sharp purges may be useful at the outset when the patient is comparatively robust, free purging is a fatal device when the patient is enfeebled, and in any case is to be deprecated as a continued treatment; that diuretics are constantly futile; but that restriction of fluid, although injurious in renal dropsy, is of some value in hepatic cirrhosis. The only reliable method is to draw off the fluid by mechanical means, and this should not be delayed. With proper precautions the risk of peritonitis from paracentesis is extremely slight, and, as a matter of personal experience, Dr. Cheadle states that in every instance of well marked hepatic cirrhosis which has done well, early and repeated paracentesis has formed an essential part of the treatment, and even in the least favourable form, that of atrophic cirrhosis, where the good effected can but be transitory, the early removal of the fluid is useful, enabling the patient to live longer and in comparative comfort. It must be pointed out that tapping in the very last stage frequently hastens the end, and that a single paracentesis without repetition, or only repeated when symptoms again become urgent, is of little service. By repeated tapping, however, combined with the use of iodide of potassium, and of digitalis in some cases, a good result may be anticipated in a fair proportion of cases of ascites.

